# Determination of Real-Time Efflux Phenotypes in *Escherichia coli* AcrB Binding Pocket Phenylalanine Mutants Using a 1,2′-Dinaphthylamine Efflux Assay

**DOI:** 10.1371/journal.pone.0021196

**Published:** 2011-06-15

**Authors:** Jürgen A. Bohnert, Sabine Schuster, Magdalena Szymaniak-Vits, Winfried V. Kern

**Affiliations:** Department of Medicine, Center for Infectious Diseases and Travel Medicine, Albert-Ludwigs-University, University Hospital, Freiburg, Germany; Texas A&M, United States of America

## Abstract

To evaluate the importance of phenylalanine residues for substrate transport in the *Escherichia coli* efflux pump protein AcrB, we subjected Phe-to-Ala binding pocket mutants to a real-time efflux assay with the novel near-infrared lipophilic membrane probe 1,2′-dinaphthylamine (1,2′-DNA). All mutations, with the exception of F617A, led to considerable retardation of efflux. F610A was the point mutation with the most pronounced impact, followed by F628A, F615A, F136A, and F178A. This is the first study to demonstrate the importance of single phenylalanine residues within the AcrB binding pocket for real-time substrate transport.

## Introduction

The *Escherichia coli* AcrB multidrug resistance (MDR) efflux pump is a member of the resistance-nodulation-division (RND) family and extrudes a wide variety of structurally diverse dyes and antibiotics [1], [2]. AcrB is part of a tripartite efflux machinery and cooperates with the membrane fusion protein AcrA and the TolC outer membrane protein.

AcrB X-Ray crystallography revealed an asymmetric trimer structure, where each protomer was suggested to correspond to a distinct functional state [3]–[5]. This led to the proposal of a 3-step transport cycle model in which substrates bind to a hydrophobic pocket, defined by phenylalanines 136, 178, 610, 615, 617, and 628, and are subsequently squeezed out towards the TolC funnel by a peristaltic-pump-like mechanism.

One of these studies could also show binding of minocycline and doxorubicin to different residues of the AcrB binding pocket [3]. Interestingly, most of the binding interactions were mediated by some of the above described phenylalanines. Minocycline interacted with F178 and F615 and doxorubicin with F615 and F617, respectively. Hence, it was proposed that the extremely broad substrate spectrum of AcrB was mainly due to the flexible interactions of various ligands with hydrophobic phenylalanines in the binding pocket.

The importance of binding pocket phenylalanines in substrate binding is also supported by several mutational studies. A study on mutations in the *Pseudomonas aeruginosa* MexD pump demonstrated that the F608C mutation (homologous to F610C in AcrB) abolished the ability of the strain to transport pyronin Y [6].

Another study, concerned with the *E. coli* MdtF (YhiV) pump, showed that the binding pocket mutation V610F (homologous to V612F in AcrB) increased the linezolid MIC by 16-fold [7]. Moreover, it has recently been shown that replacing single binding pocket phenylalanines with alanine markedly reduces various substrate MICs, with the most effective mutation being F610A [8]. It seems that this phenotypic change results mostly from altered substrate recognition, although it cannot be ruled out that the phenotype is at least partly due to moderately lower (about 43% as determined by Western blot densitometric quantification) AcrB expression, compared with the F628F control strain.

While the F610A mutation affects most substrate MICs (with the exception of linezolid and Hoechst 33342), it does not markedly influence the accumulation of ethidium and phenylalanine-arginine ß-naphthylamide (PAßN) in fluorescence-based assays. Instead, the most effective mutation in these accumulation assays was found to be F628A. The reason for this discrepancy remains unclear.

All these mutational studies have so far dealt with phenotypic characterization of the strains using dye accumulation assays and MIC microdilution assays which give only an indirect and rough estimation of the efflux capacity. The above methods ignore the contribution of influx to the overall accumulation of a given substrate, and this often makes a direct quantitative comparison of the impact of single mutations on substrate efflux relative to the wildtype strain difficult. In a recent paper on AcrAB-TolC transport kinetics of ß-lactams it could be demonstrated that it is impossible to derive any conclusions on whether a given compound is a “good” substrate of the efflux pump, if only MIC changes between an efflux-competent strain and its pump deletion counterpart are compared and no influx rates are determined [9]. Since efflux and influx act in synergy, a poor substrate permeability of the outer membrane will overestimate the efficiency of the efflux pump and vice versa. This also means that a direct quantitative comparison of substrate efflux rates, which would be highly desirable to derive kinetic information, including substrate competition, cannot be determined from MIC data.

If the influx rates of a substrate are not known or cannot be determined easily, the only other solution to determine the efficiency of substrate efflux would be to use a real-time efflux assay where efflux is triggered in bacterial cells preloaded with a substrate and efflux can be measured directly as a function of time. Such an assay has been recently published in a highly optimized form that allowed for the direct measurement of AcrAB-TolC Nile red efflux using spectrofluorometry [10].

To overcome the above problems with indirect efflux assays in phenotypic characterization of AcrB binding pocket mutants and to examine whether the suggested central role of residue F610 in substrate recognition could be reproduced in a real-time substrate transport assay, we opted to use such an assay in our previously described AcrB binding pocket Phe-to-Ala mutants. Instead of Nile Red we used the novel dye 1,2′-dinaphthylamine (1,2′-DNA) which has properties that make it very suitable for MDR efflux research. It is very lipophilic (log *P* 6, according to http://www.sioc-ccbg.ac.cn/software/xlogp3) and like Nile red well retained in the membrane phospholipids. We chose 1,2′-DNA instead of Nile red, since the latter is less lipophilic (log *P* 3.8) and we hypothesized that since the importance of the AcrB binding pocket phenylalanines in substrate recognition (due to lipophilic interactions) has been well established, 1,2′-DNA might be the substrate of choice to characterize the phenotypes of single Phe-to-Ala mutants. Moreover, 1,2′-DNA has the unique property of being capable of emission in the near-infrared range of the spectrum where the cellular autofluorescence is generally very low.

## Materials and Methods

### Strains and culture conditions

The AcrAB-TolC overexpressing *E. coli* strain 3-AG100 [11] and the 3-AG100-derived AcrB Phe-to-Ala binding pocket mutants [8] have been described previously. The strains were maintained at −80°C in CRYOBANK vials (Mast group, Merseyside, UK) for cryoprotection. They were grown at 37°C in modified Lysogeny Broth (LB), containing 1% tryptone, 0.5% yeast extract, and 1% NaCl.

### MIC of 1,2′-DNA

Serial dilutions of 1,2′-DNA in LB were prepared in 96-well plates and MICs were determined by overnight cultivation of 3-AG100 in accordance with CLSI/NCCLS M100-S20 guidelines (available at http://www.clsi.org).

### 1,2′-DNA efflux assay

To determine the optimal excitation and emission wavelengths for an 1,2′-DNA efflux assay in whole cells, the AcrAB-TolC overexpressing *E. coli* strain 3-AG100 was deenergized by overnight incubation in LB and loaded with 4 µM 1,2′-DNA, obtained from TCI-Europe (Zwijndrecht, Belgium), in the presence of 5 µM carbonyl cyanide *m*-chlorophenylhydrazone (CCCP) as described previously in the Nile red efflux assay protocol [10]. The only modification was that the dye loading was performed at room temperature (OD_600 nm_ of the cells was 0.25) and the loading time reduced to 2.5 h. The emission spectra were then recorded within a 300 nm to 900 nm range, while varying the excitation wavelength of the deenergized cells at 10 nm intervals (slit width 10 nm) from 200 to 500 nm using a Perkin Elmer LS 55 spectrumfluorimeter. Thereafter, the spectral scans were repeated with energized cells (after addition of glucose to a final concentration of 50 mM). The Phe-to-Ala 3-AG100-derived AcrB binding pocket mutants were then subjected to a 1,2′-DNA efflux assay at dye loading concentrations (DLC) ranging from 0.25 µM to 64 µM (the dye was found to precipitate at higher concentrations).

To be able to compare the efflux properties of the mutants at a given DLC quantitatively (in the absence of a kinetic model), we determined the efflux half time (EHT), which is the time needed for a reduction of 50% of the dye fluorescence after energization (over a time course of 400 s).

## Results and Discussion

### 1,2′-DNA MICs

Using MIC microdilution assays we established that 1,2′-DNA was non-toxic to the cells at concentrations of up to 64 µM (higher concentrations led to precipitation of dye).

### Determination of fluorescent properties of 1,2′-DNA

1,2′-DNA is an environment-sensitive membrane probe like the well-known structurally related N-phenyl-1-naphtylamine or NPN (almost non-fluorescent in aequous solution but strongly fluorescent upon partitioning into the phospholipid bilayer), which has already been used in an AcrAB-TolC efflux assay [12]. However, in the latter study high concentrations of CCCP (100 µM) were used to preload the cells with NPN. This normally leads to marked retardation of dye efflux after energization, since some CCCP is retained in the membranes even after repeated washing, as described in the Nile red efflux study [10].

We established that the highest fluorescence intensity ratio between the deenergized and energized state in the near-infrared band (∼6-fold) could be obtained using an excitation wavelength of 370 nm and an emission wavelength of 810 nm. The signal-to-background ratio was ∼10. Apart from the near-infrared emission maximum at 810 nm, another maximum could be detected at 415 nm, similar to NPN, which has an emission maximum at 420 nm, but does not fluoresce in the near-infrared band. The only other near-infrared environment-sensitive lipophilic membrane probe we are aware of is the carbocyanine dye 1,1′-dioctadecyl-3,3,3′,3′-tetramethylindotricarbocyanine iodide (DiR) [13] which in comparison with 1,2′-DNA has a smaller Stokes shift and lower emission maximum in the near-infrared band (absorption and emission maxima at 750 nm and 782 nm, respectively).

The novel membrane probe 1,2′-DNA is an excellent dye for membrane and membrane transport research due to the second emission maximum within the near-infrared band of the light spectrum (810 nm) where low cellular autofluorescence and low interference with substrates or competitors is generally encountered. Another advantage is that the dye is - like Nile red - well retained in the cell envelope while the bacteria are in the deenergized state, but is rapidly mobilized by the AcrAB-TolC efflux system upon energization.

We hypothesize that 1,2′-DNA could also be used well for quenching or fluorescence resonance energy transfer (FRET) studies involving membranes or membrane proteins due to its unique properties.

### Efflux phenotpyes of binding pocket mutants

**Figure 1:**
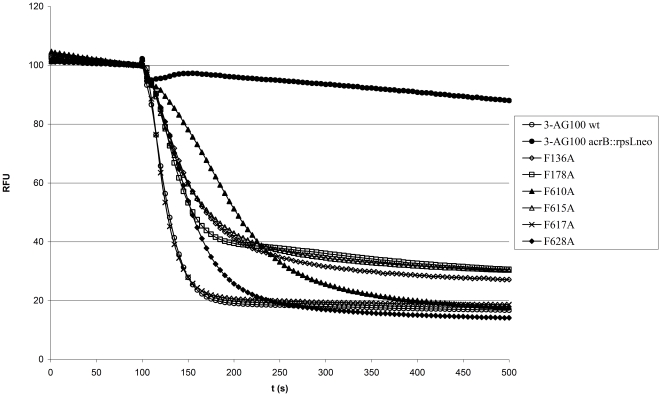
Representative 1,2′-DNA efflux curves of 3-AG100-derived AcrB binding pocket mutants. After preloading with 4 µM 1,2′-DNA the cells were energized at 100 s with 50 mM glucose. Fluorescence intensity is given as relative fluorescence units (RFU) with preenergization levels adjusted to 100 RFU.

Interestingly, all mutations, except F617A, led to a considerable retardation of real-time 1,2′-DNA efflux (in the order F610A, F628A, F615A, F136A, F178A from high impact to low impact) regardless of the dye concentration used (Table 1 and Fig. 1). The F628F pseudomutant, which was generated to demonstrate that the site-directed mutagenesis protocol introduced no systematic phenotypic changes, behaved like the 3-AG100 AcrB wildtype strain in the efflux assays (data not shown). Efflux was abolished in the *acrB* deleted control strain 3-AG100 *acrB*:: *rpsLneo* (EHT>400 s).

**Table 1:** Efflux half times (EHT) in AcrB binding pocket mutants derived from 1,2′-DNA efflux assays over a duration of 400 s at different dye loading concentrations.

Strain	EHT (s) at 1,2′-DNA loading concentration									
	0.25 µM		1 µM		4 µM		16 µM		64 µM	
	Mean^a^	SD	Mean	SD	Mean	SD	Mean	SD	Mean	SD
3-AG100 wt^b^	20	5.0	19	1.5	24	2.5	27	2.9	37	5.2
3-AG100 F136A	40	18.7	44	12.1	53	7.1	51	3.6	54	3.1
3-AG100 F178A	31	13.6	32	5.0	38	7.0	42	4.6	43	3.5
3-AG100 F610A	73	24.0	67	8.3	97	9.6	118	5.9	146	16.8
3-AG100 F615A	37	7.1	40	10.6	48	6.7	48	5.1	55	1.2
3-AG100 F617A	19	2.1	19	4.2	22	1.5	26	0.6	30	2.3
3-AG100 F628A	54	12.9	52	13.1	53	13.1	55	11.9	68	14.6

a All measurements were done in triplicate.

b wildtype strain for AcrB.

In our previous study on phenotypes of AcrB binding pocket mutants, the F610A mutation had the most pronounced effect on substrate MICs, but failed to demonstrate the same magnitude of phenotypic changes in our ethidium and PAßN accumulation assays [8]. In contrast, the very lipophilic 1,2′-DNA clearly demonstrated the dominant role of the F610 residue in AcrB substrate efflux, in line with the previous MIC assays. We can only hypothesize that the observed discrepancy between the assays might be specific to ethidium and PAßN - probably due to their structural properties.

**Figure 2:**
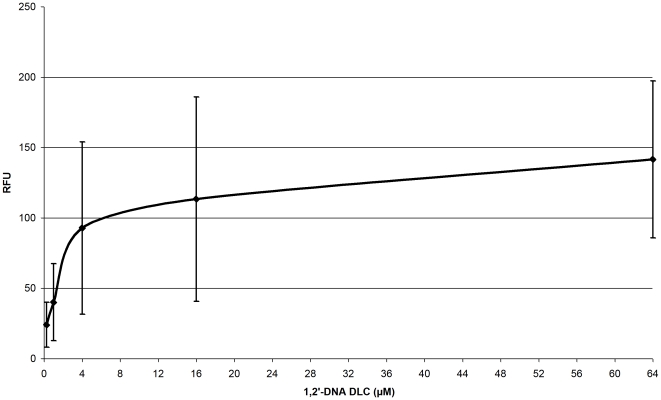
Increased quenching of 1,2′-DNA fluorescence at higher dye loading concentrations (DLCs) in strain 3-AG100. Depicted are relative fluorescence units (RFU) of deenergized cells preloaded with 1,2′-DNA after resuspension in fresh PPB. All experiments were done in triplicate. The error bars show the standard deviation.

To compare the efflux efficiency of different mutants at a given DLC (using a concentration range from 0.25 µM to 64 µM), we derived the efflux half time (EHT), which is the time needed for the fluorescence to drop to 50% after energization with glucose over a time period of 400 s (Table 1). We used this descriptor since we could not derive Michaelis-Menten based kinetic constants, due to the fact that the relationship between the DLC and the preenergization relative fluorescence units (RFU) was not a linear one (Fig. 2), but rather followed an exponential function (with higher DLCs giving a dramatically lower increase in fluorescence intensity). There might be two possibilities that explain this observation: first, the phenomenon might be due to increased self-quenching or second, it might be due to membrane saturation effects. In either case the calculation of initial rates, normally used for the determination of kinetic constants, would lead to a dramatic error at higher DLCs.

Likewise, the EHT descriptor - although in theory somewhat more robust to the above described RFU/DLC non-linearity than initial rates - cannot be used to draw any conclusion about the transport saturation characteristics of the AcrAB-TolC system, if the exact amount of self-quenching or membrane saturation effects is not known.

Nevertheless, this parameter is still useful to quantitatively compare the efflux efficiency of different mutants if the same DLC is used as a basis of comparison, since the preenergization RFU starting point (which is related to the intracellular dye concentration) should in this case be the same for all mutants. Using our experimental data, it can be demonstrated that the ratio between the EHT for a given mutant and the EHT for the AcrB wildtype strain is relatively constant over a wide concentration range (Table 1). For instance, the EHT is 73 s at a DLC of 0.25 µM in the F610A mutant and 20 s in the AcrB wildtype strain, respectively. The ratio is thus 73 s/20 s = 3.7. The EHT ratio at the higher DLC of 64 µM amounts then to 146 s/37 s = 3.9.

If one assumes that the observed RFU/DLC non-linearity is entirely due to dye self-quenching (which is currently unclear), and one linearly reconstructs the self-quenching-adjusted fluorescence (F_sqa_) curves using an Excel exponential curve fit (leading to the equation F_sqa_(t) = 12e^0.039F(t)^, where F stands for the experimentally determined RFU value) one can demonstrate that the EHT would not increase up to a DLC of 64 µM. In the given example the calculated EHTs at 64 µM would be 66 s for 3-AG100 F610A and 18 s for 3-AG100 (giving a ratio of 3.7 which is similar to the above described cases, again demonstrating the robustness of such an EHT ratio even under conditions of extreme self-quenching).

This would mean that the transport capacity of the pump would be far from saturation and that the experimentally observed EHT increase with higher DLCs would be due to dye self-quenching artifacts.

However, such a model is currently entirely hypothetical and experimental self-quenching data would be needed to verify it. This example simply serves to illustrate that the experimentally determined concentration-dependent increase in EHTs might not necessarily reflect saturation of substrate transport but might be due to other factors related to the physical properties of the dye and/or the cell envelope.

Although lipophilic dyes like 1,2′-DNA, NPN or Nile red are currently the best tool to characterize the real-time behaviour of RND efflux pumps in whole cells, a limitation of these dyes has to be considered: their hydrophobicity limits the application to relatively low DLCs. However, using more hydrophilic dyes would lead to technical problems in a real-time efflux assay, since they would probably not be well retained in the membrane phospholipids. It would thus be interesting to generate derivates of 1,2′-DNA that are increasingly hydrophilic and to examine the amount of preenergization retainment in the membrane phospholipids.

Moreover, despite the fact that we could show that 1,2′-DNA is an excellent dye for use with the *E. coli* AcrAB-TolC system, it has to be taken into account that the activation of efflux by means of glucose addition does not work well in non-fermenting bacteria like *Pseudomonas aeruginosa*, although we have recently found that the 1,2′-DNA efflux assay can be used successfully in an *E. coli* strain expressing only the MexAB-OprM system (data not shown).

### Conclusion

We have described a novel real-time efflux assay using the excellent AcrAB-TolC substrate 1,2′-DNA. Since influx does not play a role in such an assay with dye-preloaded cells, we have been capable of directly comparing the efflux efficiency of phenylalanine point mutants over a wide concentration range. While we have not yet been able to derive a kinetic model due to a lack of knowledge about the relationship between the experimental RFU values and the intracellular dye concentration, conceiving such a model might in principle be possible if the amount of self-quenching and/or dye loading saturation could be determined experimentally.
